# Comparative Safety of Six First-Line Antiepileptic Monotherapies in Pediatric Epilepsy Using the United States FDA Adverse Event Reporting System

**DOI:** 10.7759/cureus.105027

**Published:** 2026-03-11

**Authors:** Toru Ogura, Chihiro Shiraishi

**Affiliations:** 1 Clinical Research Support Center, Mie University Hospital, Tsu, JPN; 2 Department of Pharmaceutical Sciences for Health Crisis Management, Faculty of Pharmaceutical Sciences, Fukuoka University, Fukuoka, JPN

**Keywords:** adverse events, antiepileptic drugs, pediatric epilepsy, pharmacovigilance, safety signal detection

## Abstract

Background and objective

Selecting an initial antiepileptic monotherapy in children requires careful consideration of safety, as adverse drug reactions may have long-term consequences for the developing brain. However, comparative safety information across commonly used first-line antiepileptic drugs (AEDs) and pediatric age groups remains limited, particularly under strictly defined monotherapy conditions. The objective of this study is to compare the safety patterns and adverse event (AE) reporting profiles of valproic acid, lamotrigine, levetiracetam, carbamazepine, zonisamide, and topiramate used as first-line monotherapy in children (0-14 years) with epilepsy and to assess potential age-related differences in AE reporting.

Methods

Pediatric epilepsy cases (0-14 years) reported to the United States FDA Adverse Event Reporting System (FAERS) between 2004Q1 and 2025Q4 were identified. Reports were included when one of the six target AEDs was recorded as the primary suspect drug and used as quasi-monotherapy; cases with concomitant use of predefined second-line AEDs or more than one first-line AED were excluded. AEs were grouped into nine predefined clinical categories. Reporting ORs (RORs) and adjusted RORs were estimated with valproic acid as the reference, adjusting for age, sex, and reporter country. A Bonferroni-adjusted two-sided significance level of 0.01 was applied. Age-stratified sensitivity analyses (0-1, 2-5, 6-11, and 12-14 years) were performed to explore possible age-dependent patterns.

Results

Among 3,581 pediatric epilepsy cases treated with first-line antiepileptic monotherapy, 431 received valproic acid, 436 carbamazepine, 1,025 lamotrigine, 1,365 levetiracetam, 262 topiramate, and 62 zonisamide. CNS disorders were the most frequently reported category across all drugs. Dermatologic disorders were more frequently reported with carbamazepine, lamotrigine, and zonisamide, compared with valproic acid. Hepatic disorders were relatively more frequently reported with carbamazepine and valproic acid and less frequently with levetiracetam and topiramate, whereas renal disorders were more frequently reported with zonisamide and topiramate. In age-stratified analyses, dermatologic and hepatic disorders tended to be reported less often among infants than among older children, while renal disorder signals with zonisamide were observed across all age groups; CNS and psychiatric disorders generally appeared to be reported more frequently with increasing age, although some overall signals were attenuated in subgroup analyses, likely reflecting reduced sample sizes.

Conclusions

This FAERS-based analysis suggests drug-specific and age-related differences in AE reporting patterns among six first-line AEDs used as monotherapy in pediatric epilepsy. The higher reported frequencies of dermatologic disorders with lamotrigine and carbamazepine, relatively higher hepatic disorder reporting with carbamazepine and valproic acid, and renal disorders with zonisamide and topiramate may raise risk awareness and support clinical monitoring, particularly in older children. Given the inherent limitations of spontaneous reporting data and the attenuation of several signals in age-stratified analyses, these findings should be considered hypothesis-generating safety signals that merit further evaluation and confirmation in well-designed prospective and population-based studies.

## Introduction

Epilepsy is a common neurological disorder, affecting millions worldwide and imposing a substantial burden because of its chronic course [1]. In routine clinical practice, treatment typically begins with accurate classification of seizure type and epilepsy syndrome, followed by selection of an appropriate first-line antiepileptic drug (AED) [2]. First-line monotherapy is central to the management of epilepsy, whereas second-line or adjunctive therapies are generally introduced only when seizure control remains inadequate or adverse events (AEs) are intolerable. Among AEDs, six agents, such as valproic acid, lamotrigine, levetiracetam, carbamazepine, zonisamide, and topiramate, are commonly used as first-line treatments in pediatric epilepsy [3-5].

Valproic acid is an older broad-spectrum agent widely used for both focal and generalized epilepsies. Lamotrigine is a sodium channel-blocking drug with a broad spectrum of efficacy and a relatively favorable cognitive profile, but it requires slow titration to mitigate the risk of rash. Levetiracetam, which binds to synaptic vesicle protein 2A, is often chosen because it can be titrated rapidly and has few pharmacokinetic interactions, although behavioral AEs are a concern. Carbamazepine remains a standard first-line option for focal seizures but may aggravate some generalized epilepsies and necessitate monitoring for hematologic and hyponatremic complications. Zonisamide, a sulfonamide derivative with multiple mechanisms, has broad-spectrum efficacy but may lead to weight loss, metabolic acidosis, nephrolithiasis, or oligohidrosis. Topiramate acts through several mechanisms and is effective against various seizure types, but cognitive slowing and nephrolithiasis are notable issues in children. In contrast, a number of other agents are more commonly employed as second-line treatments [3].

Pediatric patients may respond differently from adults to AEDs in terms of both efficacy and safety; therefore, delineating the safety profiles of first-line agents specifically in children is crucial [6]. However, many previous studies have permitted concomitant second-line or adjunctive AED use, complicating isolation of monotherapy effects because of potential drug-drug interactions [7,8]. Treatment complexity arising from the combined use of first- and second-line therapies underscores the importance of comparing these six major AEDs under conditions that rigorously exclude concomitant second-line drugs. At the same time, large-scale prospective trials in pediatric epilepsy remain difficult to conduct because of ethical, practical, and logistical constraints [9,10].

To address this evidence gap, the present study used the United States FDA Adverse Event Reporting System (FAERS) [11] to characterize the AE reporting patterns in children (0-14 years) treated with one of the six first-line AEDs as quasi-monotherapy, excluding cases involving concomitant second-line AED use. Although FAERS is subject to limitations such as underreporting, duplicate entries, and reporting biases, it provides valuable post-marketing pharmacovigilance data. In this context, we aimed to explore disproportional reporting signals across major AE categories, including CNS, dermatologic, hepatic, GI, hematological, psychiatric, respiratory, renal, and cardiovascular disorders, as a hypothesis-generating approach that may help inform drug selection and guide future analytical and prospective studies in pediatric epilepsy. Therefore, the aim of this study was to compare the reported AE reporting patterns and disproportionality safety signals of six first-line AEDs in pediatric epilepsy (0-14 years) using FAERS, with particular attention to age-related differences in AE reporting patterns.

## Materials and methods

Data source

Reports of AEs were obtained from FAERS, a publicly available pharmacovigilance database that has been updated quarterly since its launch in 2004. The system was originally named the Adverse Event Reporting System (AERS) before being upgraded and renamed FAERS in the fourth quarter of 2012 (2012Q4). For this study, datasets spanning 2004Q1 through 2025Q4 were downloaded from the official FAERS website on January 31, 2026. The relevant compressed data files, "aers_ascii_yyyyQq.zip" and "faers_ascii_yyyyQq.zip" (where "yyyy" denotes the year and "q" the quarter), served as the data source. Each dataset included several core tables: patient demographics (DEMO), drug information (DRUG), indications for use for the reported drugs (INDI), reported AEs (REAC), and drug therapy start-end dates (THER). Variable alignment between legacy AERS data and the later FAERS structure followed specifications in the official FAERS documentation.

In the FAERS database, submissions of additional information for the same case appeared as a new entry with an incremented {caseversion} (safety report version number) rather than overwriting the previous version. Thus, for cases sharing a common {caseid} (number for identifying an FAERS case), only the entry corresponding to the highest {caseversion} was used in analyses. The legacy AERS data, which lack the {caseversion} field, were processed using the unique identifiers {ISR} (unique number for identifying an AERS report) and {CASE} (number for identifying an AERS case) to retain only the latest record. FAERS variable names are shown in curly braces for reference.

Prior to statistical analyses, key demographic variables, such as {sex}, {age} (patient age at event), {weight}, and {reporter_country} (country of reporter), were standardized. Three AERS text files were found to contain formatting errors, such as missing line breaks at three specific locations (line 322,967 in DRUG11Q2.txt, line 247,896 in DRUG11Q3.txt, and line 446,738 in DRUG11Q4.txt). These three records, each in a different file, were manually corrected only to reinsert the missing line breaks and restore the original tabular structure, without any modification of the clinical content or variable values, to ensure data consistency.

This study used de-identified data from the publicly available FAERS and did not involve direct interaction with individual patients or access to identifiable personal information. Therefore, in accordance with institutional and national ethical guidelines, institutional review board approval and informed consent were not required for this study.

Study design

This retrospective pharmacovigilance study analyzed disproportionality signals of AEs from the FAERS database among pediatric epilepsy patients (0-14 years) receiving first-line AED monotherapy. Pediatric epilepsy cases were defined as individuals aged 0-14 years who received one of six first-line AEDs (valproic acid, lamotrigine, levetiracetam, carbamazepine, zonisamide, or topiramate) for an indication recorded in the {indi_pt} variable that matched any epilepsy-related terms listed in Appendix A. When the {prod_ai} variable (product active ingredient) was available, drugs were identified by their generic names; for earlier periods lacking {prod_ai}, both generic and brand names in the {drugname} field were used. The complete list of searched generic and brand names is provided in Appendix B.

Reports were excluded if the recorded drug administration start date {start_dt} occurred after the AE date {event_dt}. For cases with multiple therapy intervals, exclusion applied only if all intervals began after the AE date; cases with at least one treatment interval starting before the AE date were retained. To minimize potential bias from polytherapy, reports involving concomitant use of second-line agents listed in Appendix B (ethosuximide, clobazam, clonazepam, diazepam, phenobarbital, phenytoin, primidone, gabapentin, oxcarbazepine, felbamate, lacosamide, rufinamide, vigabatrin, or perampanel) were excluded. Cases involving more than one first-line AED (i.e., non-monotherapy) were also excluded. Duplicate entries, defined as records sharing identical demographics, medications, AEs, and dates, were collapsed into a single case. Only reports designating the target drug as the primary suspect ({role_cod} variable) were retained.

AEs were categorized into nine groups: CNS, dermatologic, hepatic, GI, hematological, psychiatric, respiratory, renal, and cardiovascular disorders. AEs were coded using standardized Preferred Terms from the Medical Dictionary for Regulatory Activities (MedDRA). Detailed lists of Preferred Terms for each category are provided in Appendix C.

Statistical analyses

Continuous variables were summarized using medians with first and third quartiles. Categorical variables were described as counts and reporting proportions (RPs) [12]. The RP for a given characteristic was calculated as the number of pediatric epilepsy reports exhibiting that characteristic divided by the total number of pediatric epilepsy reports in FAERS.

Differences in AE profiles among the drug groups were evaluated using reporting ORs (RORs) [13] derived from univariate binary logistic regression analysis, as well as adjusted RORs (aRORs) estimated by multivariate binary logistic regression analysis accounting for patient-level covariates ({age}, {sex}, and {reporter_country}). The {reporter_country} variable was categorized into four regions: North America, Asia, Europe, and other continents, as most reports originated from North America, Asia, and Europe. The {weight} variable was not included as an adjustment factor because of its high proportion of missing values in order to reduce bias and enhance the stability of model estimates.

For both ROR and aROR analyses, valproic acid served as the reference drug, and effect estimates were obtained for lamotrigine, levetiracetam, carbamazepine, zonisamide, and topiramate, corresponding to five primary hypothesis tests. To account for multiple testing across these five contrasts, a Bonferroni correction was applied, yielding a two-sided significance level of 0.01. Accordingly, 99% confidence intervals (CIs) were reported to match this adjusted alpha level. For covariates in the regression models, the reference categories were set to female for {sex} and “other continents” for {reporter_country}.

Although aRORs allow better control for confounding, small sample sizes due to missing covariate data led us to require both RORs and aRORs to be significant at p < 0.01. Additionally, sensitivity analyses were performed by age categories (0-1, 2-5, 6-11, and 12-14 years) to evaluate age-specific patterns in AE reporting.

In accordance with previous studies, all effect measures derived from FAERS were explicitly labeled as “reported” to emphasize that they reflect disproportionality in reported cases rather than true incidence in the source population [12,13]. Statistical analyses were conducted using R version 4.4.1 (R Foundation for Statistical Computing, Vienna, Austria).

## Results

Study population

**Figure 1:**
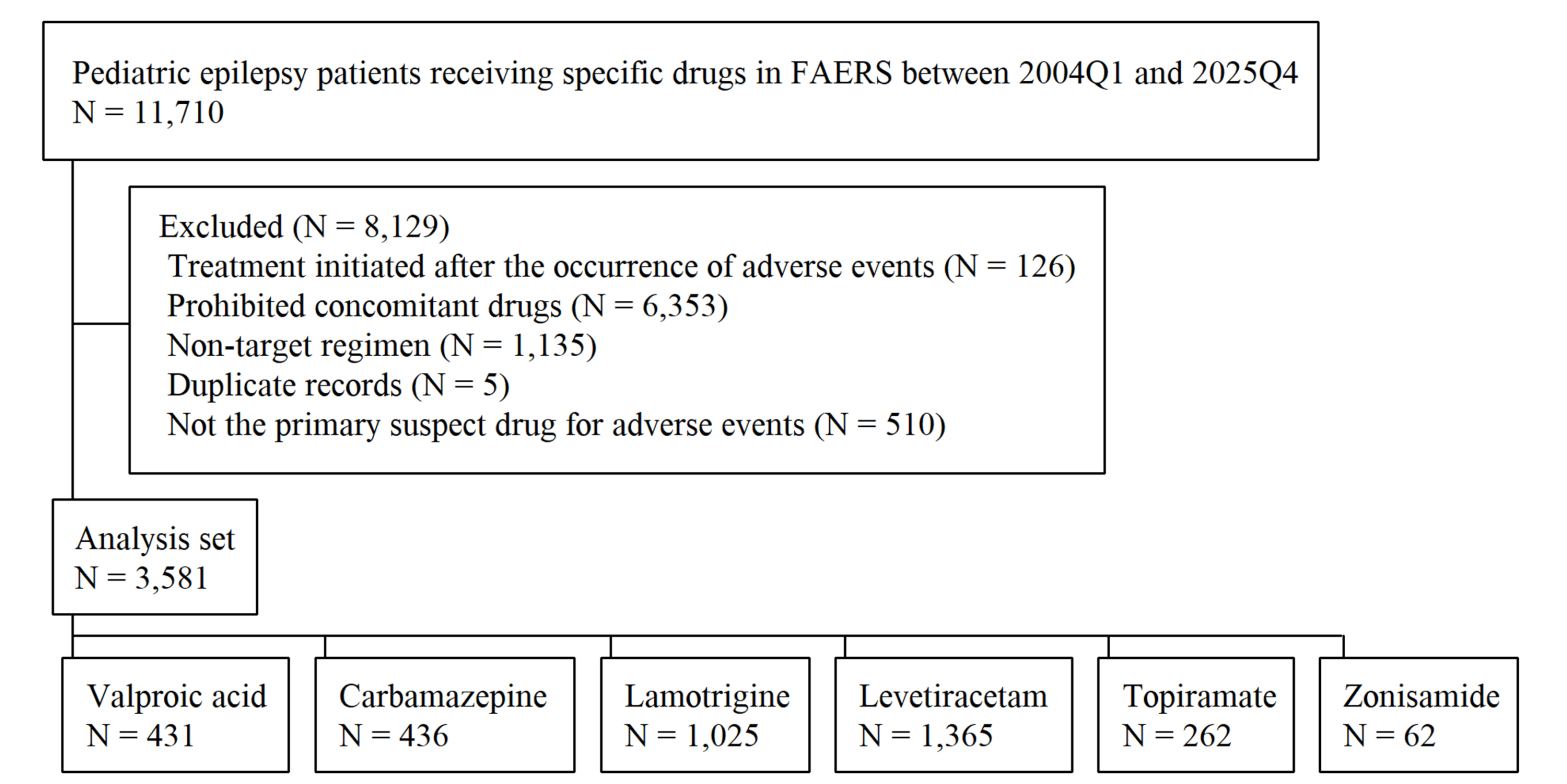
Flowchart of case selection for first-line AED monotherapy in pediatric epilepsy AED, antiepileptic drug; FAERS, FDA Adverse Event Reporting System

FAERS data between 2004Q1 and 2025Q4 identified 11,710 pediatric cases (0−14 years) receiving first-line AEDs who met initial eligibility criteria (Figure 1). After applying exclusion criteria, 8,129 cases were removed, yielding a final cohort of 3,581 cases receiving monotherapy with one of six first‑line AEDs: valproic acid (N = 431), carbamazepine (N = 436), lamotrigine (N = 1,025), levetiracetam (N = 1,365), topiramate (N = 262), and zonisamide (N = 62).

Table 1 summarizes demographic characteristics by treatment group. Sex distribution was generally balanced, with females comprising 43.6-51.5% and males 43.7-53.4% across drugs (unknown sex: 1.6-5.0%). Age distribution varied substantially: lamotrigine (32.5%) and levetiracetam (30.3%) had the highest proportions of infants (0-1 year), whereas carbamazepine (46.8%) and zonisamide (45.2%) peaked among school-age children (6-11 years). Median weight ranged from 13.0 kg (levetiracetam) to 32.0 kg (zonisamide), with weight unknown in 48.5-79.8% of cases. Reporting continent also differed: valproic acid and lamotrigine showed the highest proportions from Europe (47.8%, 54.9%), carbamazepine from Asia (40.8%), and zonisamide cases were most frequently from North America (33.9%) or Asia (32.3%) (unknown continent: 2.4-9.7%).

**Table 1:** Characteristics of pediatric epilepsy patients receiving first-line AED monotherapy Age is summarized as medians with first and third quartiles. Other variables are summarized as frequency (RP). AE, adverse event; AED, antiepileptic drug; RP, reporting proportion; Q1, first quartile; Q3, third quartile

Characteristics	Valproic acid (N = 431)	Carbamazepine (N = 436)	Lamotrigine (N = 1,025)	Levetiracetam (N = 1,365)	Topiramate (N = 262)	Zonisamide (N = 62)
Sex, n (%)						
Female	188 (43.6)	213 (48.9)	528 (51.5)	701 (51.4)	115 (43.9)	28 (45.2)
Male	230 (53.4)	213 (48.9)	471 (46.0)	596 (43.7)	135 (51.5)	33 (53.2)
Unknown	13 (3.0)	10 (2.3)	26 (2.5)	68 (5.0)	12 (4.6)	1 (1.6)
Age category, n (%)						
0-1 year	44 (10.2)	41 (9.4)	333 (32.5)	413 (30.3)	60 (22.9)	2 (3.2)
2-5 years	148 (34.3)	98 (22.5)	126 (12.3)	318 (23.3)	64 (24.4)	18 (29.0)
6-11 years	157 (36.4)	204 (46.8)	350 (34.1)	360 (26.4)	96 (36.6)	28 (45.2)
12-14 years	82 (19.0)	93 (21.3)	216 (21.1)	274 (20.1)	42 (16.0)	14 (22.6)
Weight, kg						
Median	23	26.4	17.2	13	18	32
Q1-Q3	14.8-40.0	15.0-43.2	3.0-36.9	3.2-31.8	10.4-31.0	20.5-43.7
Unknown, n (%)	344 (79.8)	296 (67.9)	523 (51.0)	713 (52.2)	127 (48.5)	42 (67.7)
Continent, n (%)						
North America	82 (19.0)	46 (10.6)	262 (25.6)	293 (21.5)	46 (17.6)	21 (33.9)
Latin America	8 (1.9)	14 (3.2)	8 (0.8)	33 (2.4)	10 (3.8)	0 (0.0)
Asia	92 (21.3)	178 (40.8)	105 (10.2)	266 (19.5)	60 (22.9)	20 (32.3)
Europe	206 (47.8)	168 (38.5)	563 (54.9)	735 (53.8)	129 (49.2)	15 (24.2)
Oceania	2 (0.5)	1 (0.2)	3 (0.3)	0 (0.0)	2 (0.8)	0 (0.0)
Africa	2 (0.5)	7 (1.6)	12 (1.2)	5 (0.4)	0 (0.0)	0 (0.0)
Unknown	39 (9.0)	22 (5.0)	72 (7.0)	33 (2.4)	15 (5.7)	6 (9.7)

AEs

Table 2 shows reporting frequencies of AE categories by first-line AED. CNS disorders were most frequent across all drugs, ranging from 22.0% (lamotrigine) to 33.4% (valproic acid). Dermatologic disorders were reported in 20.4% (lamotrigine) and 19.7% (carbamazepine) of cases versus 1.9-9.7% for other drugs. Hepatic disorders occurred in 8.1% (valproic acid) and 12.8% (carbamazepine) of cases versus 1.1-6.5% for others. GI disorders ranged from 5.4% (levetiracetam) to 11.5% (carbamazepine). Renal disorders were reported in 14.5% of zonisamide cases versus 0.2-5.3% for others.

**Table 2:** Summary of AE categories by first-line AED and age group in pediatric epilepsy Data are summarized as frequencies (RPs). For each patient, multiple occurrences of the same AE category are counted as one case within that category. If a patient experiences AEs in multiple categories, each category is counted separately. AE, adverse event; AED, antiepileptic drug; RP, reporting proportion

Age category	AE category	Valproic acid	Carbamazepine	Lamotrigine	Levetiracetam	Topiramate	Zonisamide
0-14 years (all patients)		N = 431	N = 436	N = 1,025	N = 1,365	N = 262	N = 62
	CNS disorders	144 (33.4)	114 (26.1)	225 (22.0)	449 (32.9)	64 (24.4)	19 (30.6)
	Dermatologic disorders	7 (1.6)	86 (19.7)	209 (20.4)	32 (2.3)	5 (1.9)	6 (9.7)
	Hepatic disorders	35 (8.1)	56 (12.8)	43 (4.2)	20 (1.5)	3 (1.1)	4 (6.5)
	GI disorders	45 (10.4)	50 (11.5)	77 (7.5)	74 (5.4)	19 (7.3)	6 (9.7)
	Hematological disorders	17 (3.9)	47 (10.8)	47 (4.6)	36 (2.6)	3 (1.1)	0 (0.0)
	Psychiatric disorders	34 (7.9)	24 (5.5)	80 (7.8)	117 (8.6)	21 (8.0)	4 (6.5)
	Respiratory disorders	15 (3.5)	20 (4.6)	76 (7.4)	65 (4.8)	8 (3.1)	5 (8.1)
	Renal disorders	5 (1.2)	1 (0.2)	15 (1.5)	29 (2.1)	14 (5.3)	9 (14.5)
	Cardiovascular disorders	17 (3.9)	14 (3.2)	37 (3.6)	57 (4.2)	21 (8.0)	3 (4.8)
0-1 year		N = 44	N = 41	N = 333	N = 413	N = 60	N = 2
	CNS disorders	12 (27.3)	8 (19.5)	40 (12.0)	60 (14.5)	7 (11.7)	0 (0.0)
	Dermatologic disorders	1 (2.3)	4 (9.8)	5 (1.5)	3 (0.7)	0 (0.0)	1 (50.0)
	Hepatic disorders	4 (9.1)	2 (4.9)	5 (1.5)	2 (0.5)	0 (0.0)	0 (0.0)
	GI disorders	3 (6.8)	6 (14.6)	4 (1.2)	5 (1.2)	10 (16.7)	0 (0.0)
	Hematological disorders	3 (6.8)	6 (14.6)	15 (4.5)	5 (1.2)	1 (1.7)	0 (0.0)
	Psychiatric disorders	4 (9.1)	1 (2.4)	6 (1.8)	4 (1.0)	2 (3.3)	0 (0.0)
	Respiratory disorders	3 (6.8)	0 (0.0)	64 (19.2)	31 (7.5)	4 (6.7)	0 (0.0)
	Renal disorders	2 (4.5)	0 (0.0)	9 (2.7)	14 (3.4)	5 (8.3)	0 (0.0)
	Cardiovascular disorders	4 (9.1)	1 (2.4)	32 (9.6)	34 (8.2)	9 (15.0)	0 (0.0)
2-5 years		N = 148	N = 98	N = 126	N = 318	N = 64	N = 18
	CNS disorders	46 (31.1)	28 (28.6)	40 (31.7)	122 (38.4)	15 (23.4)	6 (33.3)
	Dermatologic disorders	3 (2.0)	13 (13.3)	30 (23.8)	13 (4.1)	1 (1.6)	0 (0.0)
	Hepatic disorders	17 (11.5)	14 (14.3)	8 (6.3)	4 (1.3)	1 (1.6)	0 (0.0)
	GI disorders	17 (11.5)	12 (12.2)	12 (9.5)	17 (5.3)	0 (0.0)	1 (5.6)
	Hematological disorders	4 (2.7)	10 (10.2)	5 (4.0)	3 (0.9)	0 (0.0)	0 (0.0)
	Psychiatric disorders	12 (8.1)	5 (5.1)	23 (18.3)	22 (6.9)	4 (6.2)	2 (11.1)
	Respiratory disorders	2 (1.4)	3 (3.1)	3 (2.4)	12 (3.8)	1 (1.6)	2 (11.1)
	Renal disorders	0 (0.0)	0 (0.0)	1 (0.8)	1 (0.3)	6 (9.4)	2 (11.1)
	Cardiovascular disorders	3 (2.0)	0 (0.0)	0 (0.0)	17 (5.3)	2 (3.1)	2 (11.1)
6-11 years		N = 157	N = 204	N = 350	N = 360	N = 96	N = 28
	CNS disorders	57 (36.3)	52 (25.5)	88 (25.1)	142 (39.4)	29 (30.2)	8 (28.6)
	Dermatologic disorders	2 (1.3)	42 (20.6)	97 (27.7)	14 (3.9)	3 (3.1)	4 (14.3)
	Hepatic disorders	13 (8.3)	27 (13.2)	17 (4.9)	11 (3.1)	0 (0.0)	3 (10.7)
	GI disorders	17 (10.8)	21 (10.3)	33 (9.4)	42 (11.7)	8 (8.3)	3 (10.7)
	Hematological disorders	9 (5.7)	18 (8.8)	19 (5.4)	16 (4.4)	1 (1.0)	0 (0.0)
	Psychiatric disorders	12 (7.6)	11 (5.4)	30 (8.6)	43 (11.9)	9 (9.4)	2 (7.1)
	Respiratory disorders	8 (5.1)	14 (6.9)	7 (2.0)	6 (1.7)	2 (2.1)	2 (7.1)
	Renal disorders	2 (1.3)	1 (0.5)	4 (1.1)	1 (0.3)	3 (3.1)	4 (14.3)
	Cardiovascular disorders	4 (2.5)	11 (5.4)	2 (0.6)	2 (0.6)	8 (8.3)	1 (3.6)
12-14 years		N = 82	N = 93	N = 216	N = 274	N = 42	N = 14
	CNS disorders	29 (35.4)	26 (28.0)	57 (26.4)	125 (45.6)	13 (31.0)	5 (35.7)
	Dermatologic disorders	1 (1.2)	27 (29.0)	77 (35.6)	2 (0.7)	1 (2.4)	1 (7.1)
	Hepatic disorders	1 (1.2)	13 (14.0)	13 (6.0)	3 (1.1)	2 (4.8)	1 (7.1)
	GI disorders	8 (9.8)	11 (11.8)	28 (13.0)	10 (3.6)	1 (2.4)	2 (14.3)
	Hematological disorders	1 (1.2)	13 (14.0)	8 (3.7)	12 (4.4)	1 (2.4)	0 (0.0)
	Psychiatric disorders	6 (7.3)	7 (7.5)	21 (9.7)	48 (17.5)	6 (14.3)	0 (0.0)
	Respiratory disorders	2 (2.4)	3 (3.2)	2 (0.9)	16 (5.8)	1 (2.4)	1 (7.1)
	Renal disorders	1 (1.2)	0 (0.0)	1 (0.5)	13 (4.7)	0 (0.0)	3 (21.4)
	Cardiovascular disorders	6 (7.3)	2 (2.2)	3 (1.4)	4 (1.5)	2 (4.8)	0 (0.0)

Table 3 presents RORs and aRORs (valproic acid reference; Bonferroni-corrected p < 0.01). For CNS disorders, lamotrigine was associated with lower reporting (ROR: 0.561 (99% CI 0.404-0.777), p < 0.001; aROR: 0.536 (99% CI 0.374-0.769), p < 0.001). For hepatic disorders, lamotrigine (ROR: 0.495 (99% CI 0.270-0.908), p = 0.003; aROR: 0.501 (99% CI 0.255-0.983), p = 0.008), levetiracetam (ROR: 0.168 (99% CI 0.081-0.352), p < 0.001; aROR: 0.160 (99% CI 0.070-0.363), p < 0.001), and topiramate (ROR: 0.131 (99% CI 0.027-0.626), p = 0.001; aROR: 0.106 (99% CI 0.016-0.708), p = 0.002) showed lower reporting. Levetiracetam also showed lower GI disorder reporting (ROR: 0.492 (99% CI 0.295-0.818), p < 0.001; aROR: 0.513 (99% CI 0.296-0.891), p = 0.002). Disproportionality signals (ROR and aROR both p < 0.01) indicated higher dermatologic disorder reporting with carbamazepine (ROR: 14.883 (99% CI 5.318-41.653), p < 0.001; aROR: 15.915 (99% CI 4.743-53.409), p < 0.001), lamotrigine (ROR: 15.514 (99% CI 5.699-42.233), p < 0.001; aROR: 22.799 (99% CI 6.934-74.960), p < 0.001), and zonisamide (ROR: 6.490 (99% CI 1.479-28.480), p = 0.001; aROR: 6.468 (99% CI 1.192-35.091), p = 0.004). Carbamazepine was associated with higher hematological disorder reporting (ROR: 2.942 (99% CI 1.388-6.237), p < 0.001; aROR: 3.167 (99% CI 1.387-7.229), p < 0.001). Renal disorder reporting was relatively higher with zonisamide (ROR: 14.468 (99% CI 3.277-63.870), p < 0.001; aROR: 13.963 (99% CI 2.976-65.518), p < 0.001).

**Table 3:** ROR and aROR For ROR and aROR, the references for regimen, sex, and continent were set to valproic acid, female, and other continents, respectively. Statistical significance was defined as p < 0.01 after Bonferroni correction for multiple comparisons. “-” indicates that no events were reported in the corresponding category (ROR and aROR not estimable). AE, adverse event; aROR, adjusted reporting OR; ROR, reporting OR

AE category	Univariate analysis		Multivariate analysis	
	ROR (99% CI)	p-Value	aROR (99% CI)	p-Value
CNS disorders				
Carbamazepine	0.706 (0.480-1.037)	0.020	0.691 (0.453-1.055)	0.024
Lamotrigine	0.561 (0.404-0.777)	<0.001	0.536 (0.374-0.769)	<0.001
Levetiracetam	0.977 (0.722-1.321)	0.842	0.969 (0.694-1.351)	0.805
Topiramate	0.644 (0.409-1.015)	0.013	0.616 (0.372-1.020)	0.013
Zonisamide	0.881 (0.413-1.877)	0.665	0.895 (0.402-1.993)	0.722
Male	0.952 (0.784-1.157)	0.517	0.990 (0.802-1.223)	0.905
Age	1.070 (1.048-1.091)	<0.001	1.072 (1.049-1.096)	<0.001
North America	2.860 (0.883-9.261)	0.021	3.017 (0.916-9.930)	0.017
Asia	1.484 (0.456-4.832)	0.389	1.409 (0.426-4.665)	0.461
Europe	1.560 (0.486-5.012)	0.326	1.611 (0.493-5.266)	0.300
Dermatologic disorders				
Carbamazepine	14.883 (5.318-41.653)	<0.001	15.915 (4.743-53.409)	<0.001
Lamotrigine	15.514 (5.699-42.233)	<0.001	22.799 (6.934-74.960)	<0.001
Levetiracetam	1.454 (0.492-4.300)	0.374	2.104 (0.600-7.373)	0.127
Topiramate	1.178 (0.257-5.397)	0.781	1.342 (0.233-7.715)	0.665
Zonisamide	6.490 (1.479-28.480)	0.001	6.468 (1.192-35.091)	0.004
Male	0.761 (0.566-1.024)	0.018	0.827 (0.588-1.164)	0.152
Age	1.136 (1.100-1.174)	<0.001	1.130 (1.088-1.173)	<0.001
North America	0.458 (0.137-1.527)	0.095	0.868 (0.237-3.177)	0.778
Asia	0.804 (0.245-2.644)	0.637	2.243 (0.616-8.167)	0.107
Europe	0.375 (0.115-1.221)	0.032	1.005 (0.280-3.600)	0.993
Hepatic disorders				
Carbamazepine	1.667 (0.929-2.993)	0.024	1.581 (0.832-3.005)	0.066
Lamotrigine	0.495 (0.270-0.908)	0.003	0.501 (0.255-0.983)	0.008
Levetiracetam	0.168 (0.081-0.352)	<0.001	0.160 (0.070-0.363)	<0.001
Topiramate	0.131 (0.027-0.626)	0.001	0.106 (0.016-0.708)	0.002
Zonisamide	0.780 (0.191-3.186)	0.65	0.694 (0.137-3.514)	0.562
Male	0.630 (0.408-0.972)	0.006	0.619 (0.385-0.995)	0.009
Age	1.062 (1.017-1.109)	<0.001	1.035 (0.984-1.089)	0.079
North America	0.121 (0.033-0.447)	<0.001	0.215 (0.055-0.837)	0.004
Asia	0.370 (0.110-1.249)	0.035	0.441 (0.122-1.595)	0.101
Europe	0.176 (0.053-0.586)	<0.001	0.307 (0.087-1.088)	0.016
GI disorders				
Carbamazepine	1.111 (0.634-1.947)	0.628	1.151 (0.623-2.128)	0.555
Lamotrigine	0.697 (0.419-1.158)	0.067	0.669 (0.384-1.166)	0.062
Levetiracetam	0.492 (0.295-0.818)	<0.001	0.513 (0.296-0.891)	0.002
Topiramate	0.671 (0.321-1.400)	0.162	0.638 (0.280-1.450)	0.158
Zonisamide	0.919 (0.283-2.987)	0.854	1.071 (0.319-3.596)	0.884
Male	0.734 (0.526-1.024)	0.017	0.768 (0.538-1.098)	0.057
Age	1.064 (1.028-1.100)	<0.001	1.064 (1.024-1.104)	<0.001
North America	1.391 (0.206-9.388)	0.656	1.946 (0.284-13.343)	0.373
Asia	0.965 (0.141-6.597)	0.962	1.175 (0.169-8.167)	0.830
Europe	1.316 (0.199-8.721)	0.709	1.987 (0.295-13.389)	0.354
Hematological disorders				
Carbamazepine	2.942 (1.388-6.237)	<0.001	3.167 (1.387-7.229)	<0.001
Lamotrigine	1.170 (0.556-2.464)	0.586	1.254 (0.553-2.845)	0.476
Levetiracetam	0.660 (0.305-1.427)	0.165	0.777 (0.338-1.790)	0.437
Topiramate	0.282 (0.055-1.434)	0.045	0.115 (0.008-1.667)	0.037
Zonisamide	-	-	-	-
Male	0.797 (0.515-1.234)	0.182	0.794 (0.497-1.268)	0.205
Age	1.044 (0.999-1.092)	0.012	1.035 (0.986-1.086)	0.069
North America	0.132 (0.028-0.623)	0.001	0.211 (0.043-1.034)	0.012
Asia	0.337 (0.079-1.437)	0.053	0.410 (0.092-1.832)	0.125
Europe	0.391 (0.097-1.584)	0.084	0.627 (0.147-2.663)	0.405
Psychiatric disorders				
Carbamazepine	0.680 (0.334-1.384)	0.162	0.651 (0.301-1.405)	0.151
Lamotrigine	0.988 (0.571-1.712)	0.957	0.906 (0.498-1.648)	0.672
Levetiracetam	1.095 (0.649-1.847)	0.656	1.218 (0.694-2.138)	0.367
Topiramate	1.017 (0.483-2.144)	0.952	1.093 (0.492-2.428)	0.775
Zonisamide	0.805 (0.197-3.295)	0.692	0.910 (0.215-3.853)	0.866
Male	0.889 (0.641-1.234)	0.356	1.032 (0.729-1.461)	0.816
Age	1.100 (1.062-1.138)	<0.001	1.110 (1.070-1.152)	<0.001
North America	0.406 (0.121-1.360)	0.055	0.420 (0.121-1.460)	0.073
Asia	0.252 (0.073-0.870)	0.004	0.238 (0.066-0.861)	0.004
Europe	0.442 (0.136-1.435)	0.074	0.534 (0.158-1.803)	0.184
Respiratory disorders				
Carbamazepine	1.333 (0.543-3.272)	0.409	1.911 (0.691-5.283)	0.101
Lamotrigine	2.221 (1.056-4.671)	0.006	2.140 (0.899-5.092)	0.024
Levetiracetam	1.387 (0.654-2.941)	0.263	1.445 (0.606-3.447)	0.276
Topiramate	0.873 (0.278-2.748)	0.761	0.693 (0.168-2.854)	0.504
Zonisamide	2.433 (0.613-9.660)	0.097	5.123 (1.179-22.258)	0.004
Male	1.048 (0.711-1.544)	0.755	0.974 (0.642-1.479)	0.873
Age	0.882 (0.842-0.923)	<0.001	0.875 (0.832-0.921)	<0.001
North America	0.237 (0.054-1.038)	0.012	0.175 (0.038-0.809)	0.003
Asia	0.236 (0.054-1.037)	0.012	0.185 (0.040-0.860)	0.005
Europe	0.545 (0.135-2.192)	0.261	0.334 (0.078-1.431)	0.052
Renal disorders				
Carbamazepine	0.196 (0.012-3.308)	0.137	0.222 (0.013-3.775)	0.171
Lamotrigine	1.265 (0.332-4.825)	0.651	0.951 (0.241-3.758)	0.926
Levetiracetam	1.849 (0.527-6.491)	0.207	1.536 (0.431-5.480)	0.385
Topiramate	4.810 (1.237-18.696)	0.003	3.319 (0.793-13.883)	0.031
Zonisamide	14.468 (3.277-63.870)	<0.001	13.963 (2.976-65.518)	<0.001
Male	1.528 (0.823-2.838)	0.078	1.298 (0.663-2.541)	0.316
Age	0.955 (0.896-1.019)	0.068	0.960 (0.893-1.032)	0.150
North America	-	-	-	-
Asia	-	-	-	-
Europe	-	-	-	-
Cardiovascular disorders				
Carbamazepine	0.808 (0.314-2.082)	0.562	0.993 (0.352-2.800)	0.986
Lamotrigine	0.912 (0.422-1.969)	0.758	0.685 (0.298-1.575)	0.242
Levetiracetam	1.061 (0.513-2.194)	0.833	0.914 (0.416-2.009)	0.769
Topiramate	2.122 (0.893-5.044)	0.025	1.998 (0.772-5.173)	0.061
Zonisamide	1.238 (0.237-6.463)	0.739	2.924 (0.522-16.388)	0.109
Male	1.121 (0.727-1.728)	0.498	0.919 (0.580-1.454)	0.634
Age	0.879 (0.835-0.926)	<0.001	0.887 (0.839-0.937)	<0.001
North America	0.122 (0.026-0.582)	0.001	0.087 (0.017-0.441)	<0.001
Asia	0.063 (0.011-0.355)	<0.001	0.045 (0.008-0.266)	<0.001
Europe	0.526 (0.131-2.117)	0.235	0.340 (0.079-1.461)	0.057

Sensitivity analyses

Table 2 presents age-stratified (0-1, 2-5, 6-11, and 12-14 years) reporting frequencies of AE categories by first-line AED, which were largely consistent with the overall 0-14 years analysis. Table 4 summarizes significant disproportionality signals (both ROR and aROR p < 0.01, Bonferroni-corrected; valproic acid reference).

**Table 4:** Disproportionality signals for AE categories by age category ROR and aROR comparing each AED with valproic acid (reference) were estimated using univariate and multivariable logistic regression, respectively. <1 = both ROR and aROR were significantly <1 (p < 0.01, Bonferroni-corrected); >1 = both ROR and aROR were significantly >1 (p < 0.01, Bonferroni-corrected); N.S. = at least one of ROR and aROR was not statistically significant (including cases in which only one or neither was significant). AE, adverse event; AED, antiepileptic drug; aROR, adjusted reporting OR; ROR, reporting OR

Age category	AE category	Carbamazepine	Lamotrigine	Levetiracetam	Topiramate	Zonisamide
0-1 year	CNS disorders	N.S.	N.S.	N.S.	<1	N.S.
	Dermatologic disorders	N.S.	N.S.	N.S.	N.S.	N.S.
	Hepatic disorders	N.S.	N.S.	<1	N.S.	N.S.
	GI disorders	N.S.	<1	<1	N.S.	N.S.
	Hematological disorders	N.S.	N.S.	<1	N.S.	N.S.
	Psychiatric disorders	N.S.	<1	<1	N.S.	N.S.
	Respiratory disorders	N.S.	N.S.	N.S.	N.S.	N.S.
	Renal disorders	N.S.	N.S.	N.S.	N.S.	N.S.
	Cardiovascular disorders	N.S.	N.S.	N.S.	N.S.	N.S.
2-5 years	CNS disorders	N.S.	N.S.	N.S.	N.S.	N.S.
	Dermatologic disorders	>1	>1	N.S.	N.S.	N.S.
	Hepatic disorders	N.S.	N.S.	<1	N.S.	N.S.
	GI disorders	N.S.	N.S.	<1	N.S.	N.S.
	Hematological disorders	>1	N.S.	N.S.	N.S.	N.S.
	Psychiatric disorders	N.S.	N.S.	N.S.	N.S.	N.S.
	Respiratory disorders	N.S.	N.S.	N.S.	N.S.	>1
	Renal disorders	N.S.	N.S.	N.S.	N.S.	N.S.
	Cardiovascular disorders	N.S.	N.S.	N.S.	N.S.	N.S.
6-11 years	CNS disorders	N.S.	<1	N.S.	N.S.	N.S.
	Dermatologic disorders	>1	>1	N.S.	N.S.	>1
	Hepatic disorders	N.S.	N.S.	<1	N.S.	N.S.
	GI disorders	N.S.	N.S.	N.S.	N.S.	N.S.
	Hematological disorders	N.S.	N.S.	N.S.	N.S.	N.S.
	Psychiatric disorders	N.S.	N.S.	N.S.	N.S.	N.S.
	Respiratory disorders	N.S.	N.S.	N.S.	N.S.	N.S.
	Renal disorders	N.S.	N.S.	N.S.	N.S.	>1
	Cardiovascular disorders	N.S.	N.S.	N.S.	>1	N.S.
12-14 years	CNS disorders	N.S.	N.S.	N.S.	N.S.	N.S.
	Dermatologic disorders	>1	>1	N.S.	N.S.	N.S.
	Hepatic disorders	>1	N.S.	N.S.	N.S.	N.S.
	GI disorders	N.S.	N.S.	<1	N.S.	N.S.
	Hematological disorders	>1	N.S.	N.S.	N.S.	N.S.
	Psychiatric disorders	N.S.	N.S.	>1	N.S.	N.S.
	Respiratory disorders	N.S.	N.S.	N.S.	N.S.	N.S.
	Renal disorders	N.S.	N.S.	N.S.	N.S.	>1
	Cardiovascular disorders	N.S.	N.S.	N.S.	N.S.	N.S.

Disproportionality signals (ROR >1 and aROR >1) were observed for dermatologic disorders with carbamazepine and lamotrigine across ages 2-14 years and additionally zonisamide in 6-11 years; hematological disorders with carbamazepine in 2-5 and 12-14 years; renal disorders with zonisamide across all age groups and topiramate in 6-11 years; and hepatic disorders with carbamazepine in 12-14 years only. Signals (ROR <1 and aROR <1) were noted for topiramate with CNS disorders in 0-1 year; levetiracetam with hepatic, GI, hematological, and psychiatric disorders across multiple age groups; and lamotrigine with GI and psychiatric disorders in 0-1 year. Several overall signals were no longer significant in age-stratified analyses, likely due to reduced sample sizes within subgroups.

## Discussion

In this pharmacovigilance analysis based on FAERS, the term “safety” refers specifically to comparative patterns of reported AEs and disproportionality signals, rather than to incidence rates or definitive causal risk estimates. This FAERS-based analysis identified distinct patterns of AE reporting among six first-line AEDs used as monotherapy in pediatric epilepsy cases aged 0-14 years. CNS disorders emerged as the most frequently reported category across all agents, consistent with their known pharmacologic effects on the developing brain [14] and the nature of epilepsy itself [15]. Dermatologic disorders showed marked disproportionality with lamotrigine, carbamazepine, and zonisamide, aligning with well-established class effects involving immune-mediated hypersensitivity reactions [16]. Hepatic signals with carbamazepine and valproic acid, and renal signals with zonisamide and topiramate, also correspond to known metabolic [17] and physiologic effects of these agents [18].

Age-stratified analyses revealed patterns largely consistent with overall findings, though several signals attenuated due to reduced subgroup sample sizes. Dermatologic signals with carbamazepine and lamotrigine were evident across ages 2-14 years but absent in infants, potentially reflecting differences in immune system maturation or titration practices [19]. Renal disorder reporting with zonisamide appeared across all age groups, consistent with its carbonic anhydrase inhibitory effects leading to nephrolithiasis risk [20]. Conversely, lamotrigine and levetiracetam showed lower hepatic and GI signals, supporting their relatively favorable profiles in younger children.

These findings build on prior pharmacovigilance studies while addressing key gaps. Unlike previous analyses that often included polytherapy [7,8], our strict quasi-monotherapy definition minimized confounding from drug-drug interactions. Levetiracetam demonstrated a favorable reporting profile across multiple categories, corroborating clinical observations of its tolerability despite behavioral concerns [21]. The relatively favorable hepatic safety of newer agents versus valproic acid and carbamazepine supports careful consideration in patients with metabolic risk factors [22].

Several strengths distinguish this study. First, the strict quasi-monotherapy definition, excluding concomitant second-line AEDs and polytherapy, isolates drug-specific signals more effectively than prior pharmacovigilance analyses that permitted combination regimens. Second, the large cohort spanning all pediatric age groups (0-14 years) addresses a critical evidence gap, as prospective pediatric epilepsy trials face substantial ethical and recruitment challenges. Third, comprehensive age-stratified analyses reveal developmental patterns that are not observable in smaller datasets. Finally, rigorous statistical methods (ROR/aROR with Bonferroni correction) enhance signal validity.

Several limitations inherent to spontaneous reporting databases must be acknowledged. FAERS data are subject to underreporting, reporting biases, and a lack of denominator information, precluding incidence estimation. Confounding by indication, severity, and reporter awareness may influence patterns, particularly for well-known signals like lamotrigine rash. In addition, FAERS reports do not systematically capture epilepsy syndrome classification, seizure type, disease severity, comorbid conditions, or treatment adherence. Consequently, we could not stratify safety signals by epilepsy subtype or severity nor adjust for comorbidities or adherence, which may influence both drug selection and AE reporting patterns. The very small number of zonisamide cases (N = 62) compared with other AEDs reduces the precision and stability of the corresponding disproportionality estimates. Despite covariate adjustment, residual confounding remains possible. Age-stratified analyses also suffered from sparse data, particularly in infants, which may have attenuated some signals.

These hypothesis-generating signals warrant confirmation through prospective cohort studies or registries with validated outcomes. Comparative effectiveness trials incorporating safety endpoints, particularly dermatologic and renal monitoring protocols, would further inform first-line selection. Until then, clinicians should weigh these reporting patterns alongside seizure type, efficacy data, and individual patient factors when selecting initial monotherapy.

## Conclusions

This analysis identified distinct AE reporting patterns across six first-line AEDs used as monotherapy in 3,581 pediatric epilepsy cases, with CNS disorders predominating across all agents and disproportionality signals emerging for dermatologic disorders (lamotrigine, carbamazepine, and zonisamide), hepatic disorders (carbamazepine and valproic acid), hematological disorders (carbamazepine), and renal disorders (zonisamide and topiramate). Age-stratified analyses largely corroborated overall findings. These hypothesis-generating signals, obtained through a strict quasi-monotherapy design and comprehensive age stratification, align with known pharmacologic profiles and may inform AED selection considerations where prospective pediatric data remain limited. Spontaneous reporting limitations preclude causal inference or incidence estimation; confirmation through controlled studies is warranted.

## Appendices

Appendix A

The epilepsy cohort was defined using the following indication preferred terms from the FAERS database, presented exactly as registered in the MedDRA. Spelling and capitalization were not modified to ensure consistency with the source data:

Atypical benign partial epilepsy, Baltic myoclonic epilepsy, Benign rolandic epilepsy, Catamenial epilepsy, Complex partial epilepsy, Epilepsy, Epilepsy congenital, Epilepsy NOS, Epilepsy of infancy with migrating focal seizures, Epilepsy petit mal, Epilepsy unspecified, Epilepsy with myoclonic-atonic seizures, Focal epilepsy, Frontal lobe epilepsy, Generalised non-convulsive epilepsy, Generalized convulsive epilepsy, with intractable epilepsy, Grand mal epilepsy, Hemiconvulsion-hemiplegia-epilepsy syndrome, Idiopathic generalised epilepsy, Idiopathic partial epilepsy, Intractable epilepsy, Juvenile absence epilepsy, Juvenile myoclonic epilepsy, Lafora's myoclonic epilepsy, Myoclonic epilepsy, Myoclonic epilepsy and ragged-red fibres, Occipital lobe epilepsy, Parietal lobe epilepsy, Partial epilepsy, PCDH19 gene-related epilepsy, Petit mal epilepsy, Post stroke epilepsy, Post-traumatic epilepsy, Progressive myoclonic epilepsy, Severe myoclonic epilepsy of infancy, Sleep related hypermotor epilepsy, Symptomatic epilepsy, Temporal lobe epilepsy.

Appendix B

**Table 5:** Generic and brand names All generic and brand names are listed exactly as registered in the United States FDA Adverse Event Reporting System database. Spelling was not modified to ensure consistency with the original terminology.

Study drug status	Generic name	Brand name
Included drug	Valproic acid	Absenor, Convulex, Depakene, Depakin, Depakine, Depakote, Depalept, Deprakine, Encorate, Epilim, Episenta, Orfiril, Orlept, Stavzor, Valcote, Valpakine
	Carbamazepine	Carbatrol, Epitol, Equetro, Tegretol
	Lamotrigine	Lamictal, Motrig, Mogine, Subvenite
	Levetiracetam	Elepsia, Keppra
	Topiramate	Epitomax, Eprontia, Qudexy, Topamax, Topiragen, Trokendi
	Zonisamide	Zonegran, Zonisade, Zonicare, Zonimid, Zonisep, Zonit
Excluded drug	Ethosuximide	Emeside, Zarontin
	Clobazam	Frisium, Onfi, Sympazan, Urbanyl
	Clonazepam	Clonapam, Clonapax, Clonapik, Clonark, Clonasig, Clonatrac, Clonax, Cloneepam, Clonix, Convaclon, Epcon, Epicloz, Epitril, Epizam, Jasoclam, Klonopin, Lonazep, Onapil, Petril, Rivotril
	Diazepam	Azepam, Calmepam, Diastat, Diazemet, Diastin, Diastinex, Diastinum, Valium, Vivol, Winarin
	Phenobarbital	Epikon, Epinil, Epitan, Ergophen, Fenobarb, Luminal, Sezaby, Solfoton
	Phenytoin	Dilantin, Phenytek
	Primidone	Mysoline, Prysoline, Resimatil, Sertan, Liskantin, Mylepsinum
	Gabapentin	Gabarone, Gralise, Neurontin
	Oxcarbazepine	Oxtellar, Trileptal
	Felbamate	Felbatol, Taloxa
	Lacosamide	Motpoly XR, Vimpat
	Rufinamide	Banzel, Inovelon
	Vigabatrin	Sabril, Vigadyne, Vigafyde, Vigadrone, Vigpoder
	Perampanel	Fycompa

Appendix C

**Table 6:** Categorization of preferred terms Preferred terms from the United States FDA Adverse Event Reporting System database are presented exactly as registered in the Medical Dictionary for Regulatory Activities. Spelling and capitalization were not modified to ensure consistency with the source data.

Adverse event category	Preferred terms
Central nervous system disorders	Confusional state, Convulsion, Dizziness, Epileptic aura, Fatigue, Grand mal convulsion, Headache, Loss of consciousness, Generalised tonic-clonic seizure, Malaise, Myoclonus, Petit mal epilepsy, Seizure, Somnolence, Status epilepticus, Tremor
Dermatologic disorders	Dermatitis, Dermatitis exfoliative, Drug rash with eosinophilia and systemic symptoms, Erythema, Pruritus, Rash, Rash maculo-papular, Stevens-Johnson syndrome, Toxic epidermal necrolysis
Hepatic disorders	Acute hepatic failure, Alanine aminotransferase increased, Aspartate aminotransferase increased, Blood bilirubin increased, Drug eruption, Drug-induced liver injury, Hepatic encephalopathy, Hepatic enzyme increased, Hepatic failure, Hepatic function abnormal
Gastrointestinal disorders	Abdominal discomfort, Abdominal pain, Abdominal pain upper, Constipation, Diarrhoea, Lipase increased, Nausea, Pancreatitis, Pancreatitis acute, Vomiting
Hematological disorders	Anaemia, Blood lactate dehydrogenase increased, Coagulopathy, Eosinophilia, Leukopenia, Lymphopenia, Neutropenia, Purpura, Thrombocytopenia
Psychiatric disorders	Agitation, Aggression, Anxiety, Behaviour disorder, Cognitive disorder, Depression, Hallucination, Insomnia, Memory impairment, Psychogenic seizure
Respiratory disorders	Apnoea, Dyspnoea, Interstitial lung disease, Neonatal respiratory distress syndrome, Oxygen saturation decreased, Pneumonia, Respiratory distress, Respiratory disorder neonatal, Respiratory failure
Renal disorders	Acute kidney injury, Calculus urinary, Congenital hydronephrosis, Nephritis, Nephrolithiasis, Renal aplasia, Renal disorder, Renal failure, Renal failure acute
Cardiovascular disorders	Arrhythmia, Bradycardia, Bradycardia neonatal, Cardiac failure, Hypotension, Hypotonia, Hypotonia neonatal, Myocarditis, Pericardial effusion, Tachycardia

## References

[1] Shan T, Zhu Y, Fan H, Liu Z, Xie J, Li M, Jing S (2024). Global, regional, and national time trends in the burden of epilepsy, 1990-2019: an age-period-cohort analysis for the global burden of disease 2019 study. Front Neurol.

[2] Glauser TA, Loddenkemper T (2013). Management of childhood epilepsy. Continuum (Minneap Minn).

[3] Rosati A, De Masi S, Guerrini R (2015). Antiepileptic drug treatment in children with epilepsy. CNS Drugs.

[4] Gonzalez-Viana E, Sen A, Bonnon A, Cross JH (2022). Epilepsies in children, young people, and adults: summary of updated NICE guidance. BMJ.

[5] Tokumoto K, Terada K, Kawaguchi N (2024). Status of epilepsy care delivery and referral in clinics, hospitals, and epilepsy centers in Japan: a nationwide survey. Epilepsia Open.

[6] French JA, Staley BA (2012). AED treatment through different ages: as our brains change, should our drug choices also?. Epilepsy Curr.

[7] Arya R, Glauser TA (2013). Pharmacotherapy of focal epilepsy in children: a systematic review of approved agents. CNS Drugs.

[8] Zhang L, Li Y, Wang W, Wang C (2022). Comparative antiseizure medications of adjunctive treatment for children with drug-resistant focal-onset seizures: a systematic review and network meta-analysis. Front Pharmacol.

[9] Soul JS, Pressler R, Allen M (2019). Recommendations for the design of therapeutic trials for neonatal seizures. Pediatr Res.

[10] Kaal KJ, Aguiar M, Harrison M, McDonald PJ, Illes J (2020). The clinical research landscape of pediatric drug-resistant epilepsy. J Child Neurol.

[11] (2026). FDA Adverse Event Monitoring System (AEMS) Quarterly Data Extract Files. https://fis.fda.gov/extensions/FPD-QDE-FAERS/FPD-QDE-FAERS.html.

[12] Ogura T, Shiraishi C (2025). Comparative analysis of adverse event profiles among seven statins for hypercholesterolemia management using the United States FDA Adverse Event Reporting System. Cureus.

[13] Ogura T, Shiraishi C (2025). Comparison of adverse events among angiotensin receptor blockers in hypertension using the United States Food and Drug Administration Adverse Event Reporting System. Cureus.

[14] Kaindl AM, Asimiadou S, Manthey D, Hagen MV, Turski L, Ikonomidou C (2006). Antiepileptic drugs and the developing brain. Cell Mol Life Sci.

[15] Anwar H, Khan QU, Nadeem N, Pervaiz I, Ali M, Cheema FF (2020). Epileptic seizures. Discoveries (Craiova).

[16] Mani R, Monteleone C, Schalock PC, Truong T, Zhang XB, Wagner ML (2019). Rashes and other hypersensitivity reactions associated with antiepileptic drugs: a review of current literature. Seizure.

[17] Björnsson E (2008). Hepatotoxicity associated with antiepileptic drugs. Acta Neurol Scand.

[18] Barnett SM, Jackson AH, Rosen BA, Garb JL, Braden GL (2018). Nephrolithiasis and nephrocalcinosis from topiramate therapy in children with epilepsy. Kidney Int Rep.

[19] Anderson GD (2002). Children versus adults: pharmacokinetic and adverse-effect differences. Epilepsia.

[20] Gidal BE, Resnick T, Smith MC, Wheless JW (2024). Zonisamide: a comprehensive, updated review for the clinician. Neurol Clin Pract.

[21] French J, Edrich P, Cramer JA (2001). A systematic review of the safety profile of levetiracetam: a new antiepileptic drug. Epilepsy Res.

[22] Vidaurre J, Gedela S, Yarosz S (2017). Antiepileptic drugs and liver disease. Pediatr Neurol.

